# Beyond biomedical interventions: social vaccinology as a framework for vaccination policy and public governance

**DOI:** 10.1093/haschl/qxag132

**Published:** 2026-06-01

**Authors:** Jose Tuells

**Affiliations:** School of Health Sciences, Universidad Cardenal Herrera–CEU, CEU Universities, Elche 03203, Spain

**Keywords:** social vaccinology, vaccination policy, public governance, vaccine hesitancy, public trust, health policy, vaccination uptake, social determinants, health communication, immunization programs, governance frameworks, policy implementation

## Abstract

Despite the extraordinary scientific success of vaccines, vaccination policy continues to struggle to sustain public trust, legitimacy, and consistent uptake in many contemporary societies. Policy responses to these challenges frequently prioritize communication strategies, behavioral interventions, or stronger implementation, implicitly treating vaccination as a primarily biomedical intervention whose effectiveness depends on evidence dissemination and administrative execution. This article argues that such approaches overlook a structural limitation within vaccinology itself: the lack of an explicit framework to address vaccination as a social process embedded in public life. We propose a tripartite model distinguishing basic vaccinology, programmatic vaccinology, and social vaccinology. Social vaccinology is defined as the domain concerned with individual and collective behaviors arising from the interaction between policy design, social influences, ethical considerations, institutional trust, and public discourse. Recognizing social vaccinology does not replace scientific evidence or programmatic delivery but complements them by clarifying the social conditions under which vaccination policies succeed or fail. By reframing vaccination as both a technical and a governance challenge, this article offers a conceptual framework to support more socially robust and sustainable vaccination policies.

## Teaser

Vaccination policy has achieved extraordinary scientific success while repeatedly confronting crises of trust and legitimacy. This article argues that integrating social vaccinology is essential to move beyond technical approaches and align vaccination policy with governance, public expectations, and long-term sustainability.

Key points

- Vaccination policies often assume that scientific evidence and effective delivery are sufficient to ensure public acceptance, overlooking vaccination's social and institutional dimensions.
- Social vaccinology provides a framework for understanding vaccination as a socially embedded public practice shaped by trust, norms, ethics, and governance.
- Integrating social vaccinology into policy design can strengthen the legitimacy, resilience, and long-term effectiveness of vaccination programs.

## Introduction

Despite the extraordinary scientific success of vaccines, vaccination policy continues to face recurrent challenges of trust, legitimacy, and acceptance in many contemporary societies. Vaccination is widely recognized as one of the most effective public health interventions, responsible for major reductions in morbidity and mortality worldwide. Yet, concerns about hesitancy, uneven uptake, and public controversy persist across different contexts and over time.^1-4^

These challenges are often framed as downstream problems of communication, behavior, or implementation, to be addressed through improved messaging, behavioral interventions, or stronger administrative enforcement.^5-7^ While such approaches can be valuable, they implicitly treat vaccination as a primarily biomedical intervention whose effectiveness depends mainly on evidence dissemination and efficient delivery. This framing may overlook the broader conditions under which vaccination policies are interpreted and accepted.

Over the past 2 decades, this picture has become more nuanced. A substantial interdisciplinary literature—often described as vaccination social science—has examined these broader conditions in depth. Foundational work has emphasized the roles of trust, institutional credibility, and public reasoning in shaping health-related behaviors.^8,9^

Subsequent research has explored how vaccination is embedded in cultural practices, social relations, and historical experience, highlighting concerns about risk, uncertainty, and collective responsibility.^10-13^

More recent studies, drawing on sociology, anthropology, political science, and science and technology studies, have examined how vaccine-related attitudes and behaviors are shaped by identity, moral frameworks, media narratives, and institutional trust, particularly in the context of contemporary vaccination debates and post–COVID-19 environments.^14-18^

This body of scholarship has also informed institutional approaches to vaccination policy. Frameworks such as the World Health Organization's Behavioral and Social Drivers of Vaccination (BeSD) explicitly recognize that vaccination behavior is shaped by social and behavioral factors operating within specific policy and health system contexts.^19-21^

At the same time, these developments reflect the increasing incorporation of psychological and social insights into vaccination programs, particularly through efforts to address hesitancy, improve communication, and strengthen uptake.^20^

From a policy perspective, however, these contributions remain spread across disciplines and are not always systematically integrated into how vaccinology itself is conceptualized. Vaccinology continues to be primarily associated with the scientific development of vaccines and their translation into clinical and public health practice.^1,22-24^ As a result, while the social dimensions of vaccination are increasingly studied, they are not consistently incorporated into how vaccination policy itself is conceptualized and governed in practice.

While social and behavioral insights are increasingly recognized, they are often incorporated in response to emerging challenges—such as declining uptake, misinformation, or public controversy—rather than being consistently embedded as a core component of vaccination policy design and evaluation.^9,12,20,21^

This article does not claim to introduce the social dimensions of vaccination as a new subject of inquiry. Rather, it seeks to synthesize and systematize an extensive interdisciplinary literature within a policy-oriented conceptual framework. Specifically, we propose a tripartite model distinguishing basic vaccinology, programmatic vaccinology, and social vaccinology. By *programmatic vaccinology*, we refer to the organization and implementation of vaccination within health systems, including delivery structures, schedules, and policy instruments. We use the term *social vaccinology* to designate the domain concerned with how vaccination policies interact with social influences, ethical considerations, institutional trust, and public discourse to shape individual and collective behaviors.

In this sense, social vaccinology is not intended as a competing field, but as an integrative framework that brings together and aligns existing insights for policy application. Building on established models of health behavior, public health practice, and historical experience,^25-29^ this framework aims to clarify why technically sound vaccination policies may nevertheless falter when questions of legitimacy, proportionality, and trust are insufficiently addressed.

The principal contribution of this article is to reframe vaccination not only as a scientific and programmatic enterprise but also as a form of public governance. From this perspective, vaccination policies involve decisions about authority, consent, proportionality, and collective responsibility, and operate within contexts shaped by uncertainty, plural values, and political contestation. Contemporary work in public administration and global health governance suggests that policy effectiveness in such settings depends not only on technical capacity but also on institutional credibility, public trust, and the ability to manage disagreement and uncertainty.^30-34^ By organizing existing scholarship into a coherent conceptual model, this article aims to provide policymakers, clinicians, and researchers with a practical framework for designing vaccination strategies that are not only effective in the short term but also legitimate, resilient, and socially sustainable over time.

### Vaccination policy beyond the biomedical lens

Vaccination policy is often designed and evaluated as if vaccines were neutral biomedical tools. Within this view, their population impact is understood to depend primarily on scientific evidence, regulatory approval, and effective implementation.^1-3,34^ This framing privileges technical success over social legitimacy.

This perspective leads policymakers to interpret delayed uptake, selective acceptance, or public controversy as downstream problems linked to misinformation, limited health literacy, or communication failures.^5,6^ Policy responses therefore tend to prioritize information campaigns, technical guidance, and administrative enforcement, implicitly assuming that improved delivery will necessarily translate into acceptance.^7^

Yet, experience across different vaccines, populations, and historical periods suggests that these explanations are only partly adequate.^28,29^ Even in settings with strong scientific consensus, robust health systems, and broad access to information, vaccination programs continue to face resistance, hesitation, and erosion of trust.^2,3^ Vaccine hesitancy and selective acceptance are now recognized as persistent features of contemporary health systems rather than transient deviations, indicating that the problem is not simply one of knowledge transfer or implementation failure.

In policy terms, vaccination is a clinical intervention and a socially embedded public practice.^8,10^ It involves collective decision-making, perceptions of authority and coercion,^7^ ethical judgments about risk and responsibility, and trust in institutions that extend well beyond the clinical encounter. When vaccination policies are framed exclusively through a biomedical lens, they risk overlooking these dimensions and responding to social tensions with technical or coercive measures that may undermine legitimacy over time. This persistent gap points to a limitation in how vaccinology is commonly framed: the lack of a clearly articulated and policy-relevant framework for understanding vaccination as a social process situated in public life.^27^

Clarifying how different domains of vaccinology contribute—and where their limits lie—is essential for understanding why technically sound vaccination policies may nonetheless fail to achieve durable public acceptance.^30,31^

### Basic vaccinology: producing vaccines as scientific objects

Basic vaccinology encompasses the discovery, design, and validation of vaccines as biomedical products. It includes laboratory-based research on immunological mechanisms, antigen selection, platform development, and preclinical evaluation. Over recent decades, advances in molecular biology, genomics, and systems vaccinology have enabled increasingly precise characterization of immune responses and vaccine candidates, reinforcing the scientific sophistication of the field.^3,22^

Clinical trials represent the translational core of basic vaccinology. Through phased evaluation, they establish safety, immunogenicity, and efficacy under controlled conditions, while regulatory approval confers scientific credibility and legal legitimacy.^23^ Within this domain, success is defined by methodological rigor, reproducibility, and compliance with regulatory standards. From a scientific perspective, vaccinology stands as one of the most successful enterprises of modern medicine.^1^ From a policy standpoint, however, basic vaccinology is both indispensable and inherently limited.^3,4^ It produces vaccines as scientifically validated objects, but it does not determine how these objects will be interpreted, trusted, or valued once introduced into everyday clinical practice and public discourse. Evidence alone does not settle questions of acceptance. Decisions about whether, when, and under what conditions vaccines should be adopted are shaped by social experience, institutional trust, and collective values that extend beyond laboratory findings.^2,8,9^

Policy frameworks that assume a straightforward transition from scientific validation to public acceptance risk overlooking these dynamics. In practice, the path from efficacy to uptake is neither linear nor guaranteed. Historical experience shows that even highly effective vaccines may encounter hesitation, controversy, or delayed acceptance depending on social and institutional context.^28,29^

This limitation becomes particularly visible when vaccines move from scientific validation to organized application within health systems—the domain of programmatic vaccinology.^24^

### Programmatic vaccinology: translating vaccines into health systems

Programmatic vaccinology concerns the translation of scientifically validated vaccines into organized health system practice. It encompasses the development of vaccination schedules, recommendations formulated by advisory committees, the prioritization of target populations, and the integration of vaccines into routine care delivery. It also includes safety surveillance, pharmacovigilance, and, in some settings, compensation mechanisms for adverse events.^23,24^ In this domain, vaccination is framed as a programmatic and administrative activity, implemented through health systems and guided by evidence-based recommendations.

Policy success at this level is typically assessed through indicators such as coverage, timeliness, and epidemiological impact.^5,6^ These metrics are essential for monitoring program performance and evaluating population-level benefits. Yet, strong recommendations and well-designed delivery systems do not automatically translate into acceptance or sustained adherence. The transition from availability to uptake remains contingent on how vaccination programs are experienced and perceived in practice.^2,3^

Programmatic vaccinology therefore operates at the interface between institutions and individuals, where trust, ethical judgment, and perceptions of risk and benefit play a central role.^8,9^ It is at this level that vaccination policies are encountered, interpreted, and sometimes contested. Decisions are shaped not only by professional and public health guidance but also by social experience, moral reasoning, and confidence in the institutions that recommend and deliver vaccines.^11-13^

The limits of this approach are increasingly evident. Even when vaccines are effective, accessible, and strongly recommended, vaccination programs may encounter hesitation, selective acceptance, or resistance.^28,29^ In such situations, policy responses often focus on intensifying delivery mechanisms or strengthening communication strategies.^5-7^ While these measures can be effective, they may fail to address the broader social and institutional dynamics that shape acceptance.

Seen in this way, the challenge is not only to improve implementation but also to understand how vaccination is experienced and negotiated in public life. In this sense, it provides the structures through which vaccination is delivered, but it does not fully account for how these structures are interpreted or legitimized by the populations they are intended to serve. This gap highlights the need for a complementary analytical perspective that explicitly addresses vaccination as a social process.

### When science and implementation are not enough

From a policy perspective, persistent gaps between vaccine availability and uptake are not anomalies. They recur across vaccines, populations, and historical contexts, even when scientific evidence is strong and programmatic delivery is well established. Evidence from past vaccination efforts—including controversies surrounding pertussis vaccination and the uneven uptake of vaccines such as human papillomavirus—illustrates how scientific success and formal recommendations can coexist with public resistance, controversy, and selective acceptance.^28,29^

In policy debates, such outcomes are often interpreted as failures of communication, compliance, or implementation.^5-7^

While these factors may contribute, treating them as primary explanations risks obscuring a more fundamental issue: vaccination policies are frequently designed as if scientific validation and effective delivery were sufficient to ensure public acceptance. This assumption narrows the policy response, encouraging repeated reliance on information campaigns, incentives, or coercive measures whenever uptake falls short of expectations.

Yet, experience suggests that acceptance is not simply the downstream result of knowledge transmission or service provision. Vaccination decisions are made within social contexts shaped by trust in institutions, perceptions of fairness, and broader political and cultural dynamics.^2,8,9^

Even in settings with high levels of scientific capacity and accessible health services, vaccination programs may encounter resistance when these conditions are not aligned.

This misalignment reflects a persistent gap between how vaccination is governed and how it is experienced in practice. Policy frameworks often assume that consent will follow evidence, and that resistance represents a deviation to be corrected. In reality, consent is negotiated, contingent, and embedded in social relationships. It depends on whether policies are perceived as legitimate, proportionate, and responsive to public concerns—particularly in contexts marked by polarization, uncertainty, or declining institutional trust.^11-13,33^

When policies fail to account for these factors, technical instruments may produce short-term gains while undermining long-term legitimacy. Measures such as mandates or incentives can be effective under specific conditions, but their repeated use may generate policy fatigue, reinforce perceptions of coercion, or erode trust in public institutions.^7,21^

Taken together, these patterns suggest that neither scientific validation nor programmatic implementation alone can explain how vaccination policies succeed or fail in practice. Rather than isolated breakdowns, recurrent tensions around vaccination point to a structural limitation in how policy success is understood. Addressing this limitation requires moving beyond a purely technical or delivery-oriented perspective and engaging more explicitly with the social and institutional conditions under which vaccination is accepted, contested, and sustained over time.

### Defining social vaccinology

Social vaccinology addresses a persistent gap in how vaccination is conceptualized within policy and practice by explicitly focusing on vaccination as a social process embedded in public life. It builds on a substantial body of interdisciplinary scholarship that has examined the social, cultural, and political dimensions of vaccination, including trust in institutions, social norms, and patterns of decision-making.^2,8-13,14-18^ Rather than introducing entirely new concerns, social vaccinology provides a structured framework that brings these insights into closer alignment with policy design and governance.

Importantly, the need to articulate such a perspective has been previously noted but not fully developed. Earlier work on the evolution of vaccinology pointed to the desirability of defining a “social vaccinology” capable of integrating the scientific and social dimensions of vaccination.^27^ The present framework builds on that initial insight by offering a more explicit conceptualization and by situating it within contemporary policy challenges.

One way to approach this is to recognize that vaccination outcomes depend not only on scientific efficacy and programmatic delivery but also on how vaccines move through what has been described as a broader “uptake continuum,” including availability, accessibility, affordability, and acceptability. While existing models have emphasized these dimensions, they also highlight that uptake ultimately depends on social meaning, perceived value, and trust.^35^ Social vaccinology builds on these insights by situating vaccination decisions within the wider social and institutional environments in which they are made.

We define social vaccinology as the domain concerned with the individual and collective behaviors that arise from the interaction between vaccination policies, social influences, ethical considerations, institutional trust, and public discourse. This definition shifts attention from attitudes alone to observable practices and from individual decision-making to the broader social processes through which vaccination is negotiated and sustained. It also recognizes vaccination as a social action shaped by relationships, authority, and collective expectations.^36^ This perspective also emphasizes the interaction between the technical sphere of expertise and the social sphere of public life, where vaccination is ultimately interpreted and acted upon.

From this perspective, vaccination decisions are influenced not only by clinical recommendation or programmatic access, but by how individuals relate to different forms of authority, both institutional and interpersonal. Empirical studies have shown that trust in healthcare professionals, as well as the influence of social networks and community norms, can significantly shape vaccination behavior.^11-13,36^

These dynamics show that acceptance is not simply a response to information but a socially mediated process. Social vaccinology therefore complements basic and programmatic vaccinology by addressing dimensions that those domains cannot fully capture. While basic vaccinology establishes the scientific validity of vaccines and programmatic vaccinology organizes their delivery through health systems, social vaccinology focuses on how vaccination policies are interpreted, contested, and legitimized in practice. It provides a framework for understanding why technically sound policies may encounter resistance or fail to achieve durable acceptance, even when they are scientifically justified and effectively implemented.

Importantly, this perspective does not assume that hesitancy or contestation can be reduced to misunderstanding or noncompliance. Instead, it treats such responses as meaningful expressions of how vaccination policies are experienced in specific social contexts. This approach aligns with broader shifts in public health and social science that emphasize the role of trust, legitimacy, and social relationships in shaping health behaviors.^2,8,9,33^

By articulating social vaccinology as an explicit analytical domain, this framework helps move vaccination policy beyond reactive responses to hesitancy toward a more systematic and anticipatory approach. It enables policymakers to consider not only how vaccines are developed and delivered but also how they are received, interpreted, and negotiated within the complex social environments in which vaccination ultimately takes place.

### Core dimensions of social vaccinology

To be analytically useful, social vaccinology can be understood as a set of interconnected dimensions that shape how vaccination policies are experienced, interpreted, and ultimately translated into behavior. These dimensions do not operate in isolation but interact dynamically across different social contexts and policy settings.^20^

A first dimension concerns institutional trust and legitimacy. Vaccination policies are more likely to be accepted when the institutions that design and implement them are perceived as credible, transparent, and accountable. Trust extends beyond confidence in scientific evidence and includes perceptions of integrity, fairness, and intent in the actions of health authorities and governing bodies.^2,8,9,33^

A second dimension relates to how risks and benefits are perceived. Individuals and communities do not evaluate vaccines solely on the basis of epidemiological data but through interpretations shaped by personal experience, cultural narratives, and information environments. These perceptions influence how vaccination is valued in relation to other concerns, including safety, necessity, and perceived urgency.^2,3,10^

Another important dimension involves social norms and collective expectations. Vaccination behaviors are embedded in social relationships and influenced by what is seen as acceptable, typical, or expected within a community. These norms are reinforced through interpersonal networks, professional authority, and broader cultural frameworks, contributing to patterns of both acceptance and resistance^11-13,15^

A further dimension concerns policy design and lived experience. Vaccination policies are not simply technical instruments but also social interventions that convey implicit judgments about responsibility, fairness, and inclusion. How policies are implemented—whether through recommendation, facilitation, incentive, or mandate—shapes how they are experienced and whether they are perceived as legitimate or coercive.^5-7,21^

Taken together, these dimensions highlight that vaccination outcomes emerge from the interaction between technical systems and social environments. Social vaccinology offers a way to analyze these interactions more systematically, helping policymakers anticipate potential points of tension and design strategies that are not only effective but also legitimate and socially sustainable.

### Implications for vaccination policy

Reframing vaccination as a social and governance process has clear implications for how policies are designed, implemented, and evaluated. If vaccination outcomes are shaped not only by scientific evidence and programmatic delivery but also by trust, perception, and social context, then policy approaches need to move beyond a primarily technical orientation and engage more explicitly with the conditions under which vaccination policies are interpreted and accepted.

A central implication concerns the role of legitimacy. Vaccination policies may be scientifically robust and operationally effective, yet struggle to achieve sustained acceptance if they are perceived as opaque, disproportionate, or insufficiently responsive to public concerns. Legitimacy depends not only on what decisions are made, but on how they are justified, communicated, and embedded in institutional practice. In this sense, trust is not simply a prerequisite for uptake but an outcome of how governance is exercised over time.^2,8,9,33^

At the same time, policy design needs to anticipate variability in how risks and benefits are interpreted across populations. Individuals do not encounter vaccination as passive recipients of evidence but as actors situated within specific cultural and informational environments. Past experiences with health systems, exposure to media narratives, and broader social conditions all shape how vaccines are perceived and valued. As a result, approaches that appear technically coherent at the population level may produce uneven outcomes in practice, particularly in contexts marked by inequality or marginalization.^2,3,10,21,35^

Vaccination policies also operate within social environments where behavior is mediated by relationships. Decisions are rarely made in isolation; they are influenced by interactions with healthcare professionals, family members, and community networks, as well as by wider patterns of social recognition and trust. Policy strategies that focus exclusively on information provision may therefore be insufficient if they fail to engage these relational dimensions. Interventions that strengthen trusted channels of communication and acknowledge the social embeddedness of decision-making are more likely to achieve sustained impact.^11-13,17^

A further implication concerns the cumulative and path-dependent effects of policy instruments. Measures such as mandates, incentives, or targeted campaigns can be effective under specific conditions, but their longer-term consequences depend on how they are perceived and experienced. Policies that rely heavily on coercive or instrumental approaches may achieve short-term improvements in uptake while simultaneously generating resistance, fatigue, or erosion of trust. Understanding these dynamics requires viewing vaccination policies not as isolated interventions but as part of an evolving relationship between institutions and populations.^5-7^

Finally, the evaluation of vaccination policies should extend beyond coverage rates and epidemiological indicators to include measures of trust, perceived legitimacy, and public engagement. These dimensions are not secondary outcomes but integral components of policy performance. Incorporating them into monitoring frameworks allows for a more nuanced understanding of success and helps identify emerging tensions before they translate into declines in uptake.

These considerations point to a broader understanding of what makes vaccination policy successful—one that goes beyond scientific robustness and programmatic efficiency to include social legitimacy and institutional trust. In practice, social vaccinology offers a framework for approaching vaccination policy in a more anticipatory and adaptive way, particularly in complex and evolving social environments.

### Why this matters now

The need to rethink vaccination policy in social terms has become especially visible in recent years. The COVID-19 pandemic brought vaccination to the center of public debate across many societies, exposing both the strengths and the limits of existing policy approaches. Despite unprecedented scientific advances and rapid vaccine development, many countries experienced uneven uptake, public controversy, and sustained challenges to trust.^2,4^

These dynamics did not emerge in isolation but made visible structural tensions long present in vaccination policy. Issues related to legitimacy, institutional trust, and public acceptance—often treated as secondary to scientific and programmatic considerations—became central determinants of policy outcomes.^2,9^

At the same time, the broader informational environment in which vaccination decisions are made has become more complex. The expansion of digital media has increased the speed and reach of both accurate information and misinformation, amplifying the role of perception, identity, and trust in shaping public responses.^18^

These developments reinforce the limitations of approaches that treat vaccination primarily as a problem of communication or implementation. They also underscore the need for frameworks that can account for how vaccination policies function within dynamic social environments characterized by plural values, uncertainty, and institutional fragility.

This highlights that social vaccinology offers a timely conceptual lens. It allows policymakers and researchers to move beyond reactive responses to hesitancy and toward a more anticipatory understanding of the conditions under which vaccination policies are likely to succeed or fail. In doing so, it supports the design of strategies that are not only scientifically sound and operationally effective but also socially legitimate and resilient over time.

## Conclusion

Vaccination has traditionally been viewed as a scientific and programmatic enterprise, grounded in biomedical knowledge and implemented through increasingly sophisticated health systems. This perspective has enabled extraordinary advances in disease prevention and remains essential to vaccine development and delivery. However, as this article has argued, it provides only a partial account of how vaccination policies function in practice and why they sometimes fail to achieve sustained public acceptance.^1,2^

By bringing together insights from a wide range of interdisciplinary scholarship, this article has proposed a conceptual framework that distinguishes between basic, programmatic, and social vaccinology. Within this framework, social vaccinology does not represent a separate field, but an integrative perspective that makes explicit the social conditions under which vaccination policies are interpreted, negotiated, and sustained. It draws attention to the fact that vaccination is a biomedical intervention and a practice grounded in social contexts, shaped by trust, norms, perceptions of risk, and institutional legitimacy.^2,8-13^

This perspective helps explain a recurring paradox in contemporary vaccination policy: why technically sound and scientifically well-supported interventions may still encounter resistance, hesitation, or uneven uptake. It suggests that these outcomes are not anomalies or simple failures of communication or implementation, but reflections of deeper structural dynamics in how vaccination policies are experienced and evaluated within society.^2,3^

The implications are both conceptual and practical. Conceptually, expanding vaccinology to include its social dimension allows for a more comprehensive understanding of policy performance, one that integrates scientific effectiveness with legitimacy and public trust. Practically, it supports the development of policy strategies that are more adaptive, context-sensitive, and capable of anticipating how different populations will respond to vaccination initiatives.^21,30-32^ This shift does not diminish the importance of biomedical advances or programmatic efficiency but situates them within a broader framework of governance in which social acceptance is integral to success.

In this sense, vaccination policy can be understood as a form of public governance operating under conditions of uncertainty, plural values, and evolving social expectations. Decisions about vaccination involve not only evidence and logistics but also judgments about proportionality, responsibility, and collective risk. As recent experiences have shown, particularly during the COVID-19 pandemic, these dimensions can become central to policy outcomes in ways that are difficult to address within a purely technical framework.^2,4^

Looking ahead, the challenge for vaccination policy is not only to continue advancing scientific innovation and improving delivery systems but also to sustain legitimacy and trust over time in increasingly complex and contested social environments. This requires moving beyond reactive responses to hesitancy toward more anticipatory and integrative approaches that account for how vaccination is embedded in public life.

Social vaccinology offers a framework for meeting this challenge. By making visible the social dynamics that shape vaccination outcomes, it contributes to a more complete understanding of how vaccination policies succeed or fail—and, ultimately, to the development of strategies that are not only effective in the short term but also resilient, legitimate, and socially sustainable over the long term.

## Supplementary Material

qxag132_Supplementary_Data
